# The Development of a Three-Dimensional Platform for Patient-Derived Ovarian Cancer Tissue Models: A Systematic Literature Review

**DOI:** 10.3390/cancers14225628

**Published:** 2022-11-16

**Authors:** Lusine Sevinyan, Priyanka Gupta, Eirini Velliou, Thumuluru Kavitha Madhuri

**Affiliations:** 1 Department of Gynaecological Oncology, Royal Surrey NHS Foundation Trust, Guildford GU2 7XX, UK; 2 Cancer Research, School of Applied Sciences, University of Brighton, Brighton BN2 4HQ, UK; 3 Centre for 3D Models of Health and Disease, Division of Surgery and Interventional Science, University College London, London WC1E 6BT, UK; 4 Bioprocess and Biochemical Engineering Group (BioProChem), Department of Chemical and Process Engineering, University of Surrey, Guildford GU2 7XH, UK

**Keywords:** scaffolds, 3D, three-dimensional, model, ovarian, cancer, patient-derived, personalised

## Abstract

**Simple Summary:**

The aim of this systematic review is to provide an overview of the state of the art on in vitro models for ovarian cancer studies, with focus on patient derived studies, which provides a personalised approach to treatment of patients with ovarian cancer.

**Abstract:**

There is an unmet biomedical need for ex vivo tumour models that would predict drug responses and in turn help determine treatment regimens and potentially predict resistance before clinical studies. Research has shown that three dimensional models of ovarian cancer (OvCa) are more realistic than two dimensional in vitro systems as they are able to capture patient in vivo conditions in more accurate manner. The vast majority of studies aiming to recapitulate the ovarian tumour morphology, behaviors, and study chemotherapy responses have been using ovarian cancer cell lines. However, despite the advantages of utilising cancer cell lines to set up a platform, they are not as informative as systems applying patient derived cells, as cell lines are not able to recapitulate differences between each individual patient characteristics. In this review we discussed the most recent advances in the creation of 3D ovarian cancer models that have used patient derived material, the challenges to overcome and future applications.

## 1. Introduction

Worldwide there were nearly 300,000 cases of OvCa diagnosed in 2018 with the 5-year survival between 30–50% despite the advances in diagnostics and treatment. In the UK alone, there were 7300 new cases reported in 2017 with 4200 deaths in 2018, and the numbers continue to steadily increase [1]. The most common type of ovarian malignancy is epithelial ovarian cancer (EOC) most of which is represented by the serous subtype [2].

The current standard for treatment of EOC is cytoreductive surgery combined with platinum-based chemotherapy [3]. In patients diagnosed with EOC where upfront surgery is medically contraindicated (e.g., comorbidities or poor performance status), or where complete cytoreduction cannot be achieved, neoadjuvant chemotherapy prior to interval debulking surgery, and adjuvant chemotherapy is an alternative therapeutic option [4]. However approximately 80% of patients will relapse following first-line chemotherapy [3]. Moreover, despite treatment, most patients appear to develop a recurrent disease which is platinum resistant, partially due to the complex and heterogeneous tumour microenvironment. Many chemotherapy agents then fail to have sustained efficacy in clinical practice, As a result, there is an unmet biomedical need for ex vivo tumour models that would predict drug responses and in turn help determine treatment regimens and possibly predict resistance before clinical studies [5].

There have been attempts to create patient-derived OvCa models since the first report by Griffon et al., in 1995 [6]. In the last decades, a number of studies [7,8,9,10,11,12,13,14,15,16,17,18,19,20,21,22,23,24,25,26,27,28,29,30,31,32,33,34,35,36,37,38,39,40,41] have been conducted aiming to create in vitro tumour models, which could be a platform for OvCa research and drug testing for specific patients prior to treatment, thereby moving towards a more personalised/individualised treatment. For the latter to be achieved efficiently, accurate in vitro models with high predicting capability are required. Such models should simulate ex vivo the OvCa evolution and response to treatment maintaining each patient’s phenotypic and genetic characteristics. The aim of this systematic review is to provide an overview of the state of the art on in vitro models for OvCa studies, with focus on patient derived studies.

### 1.1. Ovarian Cancer Tumour Characteristics

In order to create a model of OvCa that would represent the in vivo environment and reflect the processes happening in the body it is important to understand the general morphology and physiology of OvCa tumours and the way it spreads.

It is well established that ovarian tumours consist of mixture of epithelial, stromal, immune, and endothelial cells which form the complex tumour microenvironment [42,43,44,45]. In their recent review Horst et al., have discussed in detail the impact and the role of heterogenous cellular components on the extracellular matrix [46]. And as outlined by Lengyel et al., this heterogeneity of cell types likely impacts tumour histology, growth potential and ability to evade chemotherapy [42]. Hence it is important to acknowledge this when creating the ovarian tumour ex vivo model.

The primary microenvironment for the ovarian carcinoma cell is the mesothelium [43,47]. An intact mesothelial cell layer can very efficiently inhibit the invasion of OvCa cells, suggesting that mesothelial cells can delay OvCa attachment and invasion [48]. There is also a pool of evidence suggesting that adipocytes play a crucial role in creating tumour microenvironment and promoting metastasis [49,50].

Fibronectin as well as integrins play an important role in spheroid growth and the abundant presence of several isoforms of fibronectin in malignant ascites suggests the importance of the microenvironment in OvCa metastasis [43]. Furuya outlines the importance of extracellular matrix, stroma and omental adipose tissue in the development of the OvCa [51]. Other sources highlight the role of fibroblasts in the OvCa growth, adhesion and invasiveness [52].

The unique anatomical location of the ovary renders OvCa cells the ability to easily metastasise in comparison to other cancers. Once the cancer cells have detached as single cells or clusters from the primary tumour, it is thought that they metastasise through a passive mechanism, carried by the physiological movement of peritoneal fluid to the peritoneum and omentum.

It is not entirely clear whether single cells detach and then aggregate to form spheroids, or if the cells detach as cell clumps that stay together while floating in ascites [43]. The other major mechanism of metastasis of the OvCa is via haematological route, which requires intra- and extravasation of the cancer cells [53].

It is also necessary to consider when creating a tumour model that in order for the tumour to grow over a certain size (>1–2 mm) a process of neovascularisation has to occur [54]. However the role of angiogenesis in OvCa development remains unclear and as mentioned by Duncan et al. [55] there are contradictory studies with regards to the influence of microvessel density in OvCa prognosis [56,57,58]. Worzfeld et al., also outline the possible role of extracellular microvesicles in invasion and metastasis and their contribution to the drug-resistance in the patients with OvCa [47]. From the therapeutic perspective, it has been demonstrated that poly(ADP-ribose) polymerase (PARP) inhibition decreases angiogenesis whereas, hypoxic state and vascular endothelial growth factor receptor 3 (VEGFR3) inhibitors induce down-regulation of BRCA1/2 and RAD51, which potentiate PARP inhibitors sensitivity. However, hypoxia is also associated with hypoxia inducible factor 1 alpha (HIF1α) up-regulation, and therefore resistance to angiogenesis inhibitors. Though, PARP1 is involved in HIF1α stabilization and consequently, inhibition of PARP may prevent HIF1α accumulation that leads to targeted hypoxic-induced apoptosis [59].

It seems evident from the up-to-date scientific reports that there are certain conditions to be met for an ideal OvCa tumour model to be achieved, and these key requirements are summarised in Table 1 below.

### 1.2. Current Models

Animal models, three-dimensional (3D) models and two-dimensional (2D) cell culture are currently the most widely used methods for creating various tissue models including tumour tissues. 2D cell cultures (in T-flasks or microplates) have dominated the in vitro landscape for the last decade [60,61]. Researchers have widely used the monolayer cell model due to its easy accessibility, relative ease of use and low cost. However, this model has several limitations. Carvalho et al., stressed the inability of 2D models to recapitulate the complex nature of tumours and the influence of the surrounding tumour microenvironment (TME) [60]. Moreover as the monolayer model has a different morphology from the in vivo model considering the lack of interactions with surrounding cells and surrounding matrix, molecular differences and inability to mimic the complex TME, it would not be the primary choice for researchers to use as a drug-testing platform as this non-physiological screening approach often results in poor predictive power for drug efficacy in patients [48,61,62]. In fact, many studies have shown that 2D models have different cellular responses to the environment [63] and drug response patterns as compared to 3D systems and to in vivo studies [64,65,66,67,68,69,70,71,72,73]. Pinto et al., note in their recent review that generally most of published work around tumour cancer models is done exclusively utilising cancer cells without any other cell types, which can not closely represent the processes occurring in the complex tumour microenvironment [74].

In order to replicate genuine tumour morphology and microenvironment animal models and 3D models have been created. Currently, animal models are considered to be gold standard in pre-clinical studies. They are more realistic than 2D in vitro systems as they are able to capture patient in vivo conditions in a more accurate manner. Currently, both small and large animal models are available for OvCa. They are mostly murine models (mouse and rat), but also include hens and SCID pigs [26,72,73,75,76,77,78,79]. Although animal studies are informative, high cost, complexity of reproduction and length of development time as well as several deficiencies, that make extrapolation to human tumour biology problematic [48], makes them less attractive for researchers [61]. As a result of limitations of 2D and the animal studies it has encouraged development of three-dimensional models of tumours, including OvCa [60].

Three-dimensional models offer the potential for ex vivo research of cell to cell interactions thus making the study of the nature of tumours easier, especially where extracellular matrix cover and stromal cells are added [58,60,62,63,80,81,82,83,84,85,86,87,88,89,90]. They also can allow the real-life oxygen, nutrient and temperature distribution and more realistic drug resistance studies [61,63,91]. Additionally, it is also feasible to culture patient samples directly in 3D models allowing for a much more realistic assessment of various therapeutic methods and leading to a personalised medicine-based approach towards cancer treatment [15].

3D models of OvCa have become highly interesting for investigation as according to recent oncogenesis theories the progression of OvCa involves detachment of cancer cells from the in situ carcinoma into cell aggregates/spheroids, and further attachment to mesothelial-lined surfaces. [92] For instance, Bapat et al., were one of the first who were able to isolate spheroid aggregates from malignant ascites of a patient [93]. Thus, a 3D model of OvCa cells could morphologically resemble multicellular aggregates in cancerous ascites [22]. It has also been shown that spheroid aggregates of malignant ascites compared to 2D models are more resistant to chemotherapy treatments, including cisplatin and taxol [94,95]. This has made the 3D model of OvCa highly useful for research.

**Figure 1:**
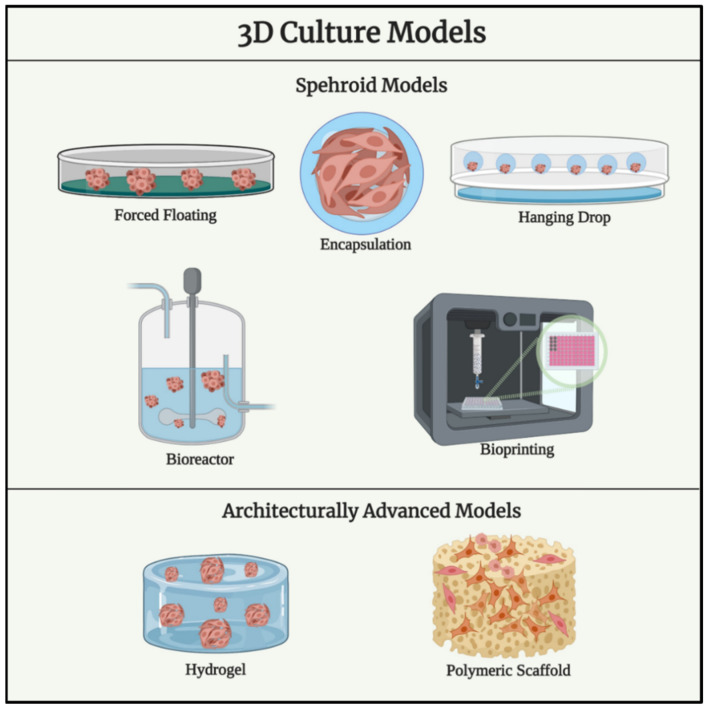
A graphical schematic of key techniques used for three-dimensional cell culture (created on BioRender.com (accessed on 14 March 2022)).

Most OvCa in vitro remodelling approaches in 3D involve, like in other diseases [63,65,66,68,96,97,98,99], (i) spheroids, (ii) hydrogel type scaffolds, (iii) synthetic highly porous polymer-based scaffolds and synthetic matrices (Figure 1).

### 1.3. Spheroid Models for Ovarian Cancer

Spheroid systems are one of the oldest and most widely used 3D cell culture set up in the field of tumour model development, drug discovery, therapeutic assessments etc. They are cell aggregates/clusters, which are formed as a result of cell-cell adhesion without attachment to culture vessel surfaces. In vitro 3D approaches have been established till date for spheroid formation like, forced floating method, hanging drop method, encapsulation-based method, 3D bioprinting and agitation method to name a few [61,100,101,102]. The close cell-cell interactions in spheroid systems assist in production of various ECM (extra cellular matrix) proteins by the cells, allowing them to form their own niche/microenvironment.

Similar to other cancer models, spheroids are also one of the most widely used system for in vitro models of OvCa and its metastasis. Ishiguro et al., have done a comprehensive overview of the representative methods for spheroid cultures of cancer cells, which include organotypic multicellular spheroids, multicellular tumour spheroids, tumour-derived organoids and tumour-derived spheroids [69]. For example, low cell number spheroids (as few as 10 cells) were created, and significant chemoresistance compared to 2D model was demonstrated by Raghavan et al. [64] Zietarska et al., were able to form spheroids up to 500 µm in size at 4 day of culture, generated from EOC cell line in hanging droplets, which allowed the researchers to have a better understanding of the cancer biology as compared to the 2D model of OvCa [21]. According to Grun et al., spheroids grown using Rotary cell Culture Systems could grow for longer periods and reach significantly higher volumes [24]. Increased chemotherapy resistance in 3D compared to 2D culture was also exhibited by Lee et al. [73] and Moraya et al. [103].

However even with extensive research of OvCa using spheroid models, they have certain inherent disadvantages. Due to their spatial characteristics, high diffusion gradient in terms of nutrients and oxygen is formed within them. This in turn results in the formation of necrotic cores at the center and decreasing cellular proliferation over time like it has been shown for other types of cancer models [61,104,105]. Spheroid systems are also difficult to maintain over long time periods (weeks or months) without re-suspending the cells to form fresh cellular aggregates. Depending on the method it is also difficult to control spheroid size and shape of the spheroids [106]. These disadvantages cannot be neglected as they can lead to differences in experimental results obtained. For instance, Lal-Nag et al., showed on modified HEY A8 cell line that spheroid size is an important consideration when comparing chemotherapeutic responses because cell metabolism, proliferation and survival vary within spheroids as they grow larger, and nutrient and oxygen gradients become pronounced [5].

### 1.4. Ovarian Cancer Models Using Biomaterials with Advances Structural Complexity

Although relatively easy to use, the disadvantages associated with spheroids models have resulted in the development of 3D cancer models with more structural complexity and stability using natural and artificial biomaterials in the form of hydrogels and polymeric scaffolds. Hydrogels are cross-linked polymeric networks consisting of a high amount of water and are able to simulate the native tissues in terms of architectural and spatial characteristics, biocompatibility and also allows nutrient and oxygen diffusion. Hydrogels can be formed of natural molecules like collagen, Matrigel and other extra cellular matrix (ECM) proteins or of synthetic materials like poly–ethylene glycol (PEG), agarose, alginate etc. Similar to other cancers, OvCa 3D models using hydrogels have been established by various groups [65,66,68,72,96,107,108]. The length of the various studies varies between 7–28 days highlighting the feasibility of relatively long-term culture in hydrogel systems. It has also been reported that hydrogel-based tumour models of OvCa shows higher resistance to chemotherapeutic agents like paclitaxel in comparison to 2D culture systems [65,66,68,96]. However, despite its multiple advantages, depending on the material properties and structural configurations (porosity, pore size, pore interconnectivity) hydrogels might not provide consistent cell distribution within them resulting in different densities of cells within them and reducing the chances of consistent spheroid formation [104]. In addition, hydrogels due to their high water content also lack mechanical strength resulting in handling difficulty [61,98,109,110]. Moreover, for natural polymers, long term culture is a difficult proposition due to high batch-to-batch variations, undefined matrix composition and restricted modification possibilities [109]. A lot of studies have been conducted to compare different types of hydrogels [65,108,109,111,112,113,114,115], however each of them were found to have certain limitations. Li et al., have done a comprehensive overview of the main types of hydrogel microenvironments discussing their pros and cons and their application for cancer research and drug screening in different types of cancer cells, and in their review they agreed that further research is necessary to focus on improvement of the modelling of tumour microenvironment and confirmation of in vitro results, and that patient-derived cells are more desirable than well established cell lines [116].

To overcome the limitations of the above methods there have been a number of developments in tissue engineering to construct polymeric scaffolds-based tumour models. Those include reports describing scaffolding systems for OvCa [16,81,117], lung cancer [117,118], pancreatic cancer [80,119,120,121,122,123,124], breast cancer [117,125], prostate cancer, melanoma [117] and others [117]. Amongst the advantages of the polymeric scaffolds, authors highlight their ability to be ‘customised’ for any type of tumour and the potential of researchers to endue scaffolds with the desired extracellular matrix properties [126]. Polymeric scaffolds are especially very promising for future research as, they incorporate the advantages of hydrogel and spheroid scaffolds, such as provision of structure, realistic spatial arrangement, realistic cell-cell and cell-ECM interactions, and also possess such qualities as good mass transfer, porosity, architectural tuneability as well as tuneable mechanical properties. However, polymeric scaffolds are more complex in terms of synthesis procedure in comparison to spheroids and hydrogels and cell retrieval can be difficult in scaffolds depending on the material used.

**Figure A1:**
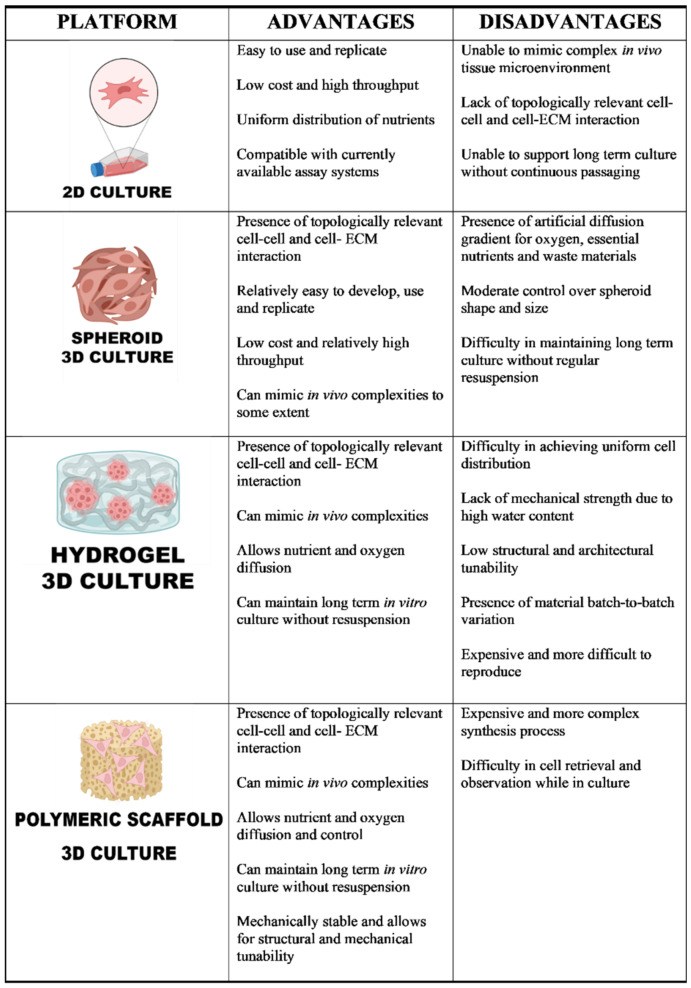
A graphical schematic of key techniques used for creation of OvCa tumour models (created on BioRender.com (accessed on 26 July 2022).

Graphic summary and comparison of different types of platforms used to replicate and research OvCa models can be found in the Appendix A (Figure A1).

### 1.5. Cell Sources in Available Ovarian Cancer In Vitro 3D Studies

The vast majority of studies aimed to research OvCa have used cancer cell lines with very few utilising patient derived samples. Despite the advantages of using cancer cell lines to set up a platform/system, i.e., availability, consistency/reproducibility, cancer cell lines are not as informative as patient derived cells, as they would not able to recapitulate differences between each individual patient characteristics, including differences in drug sensitivities due to changes in gene expression following culture and passage [127]. Furthermore, they have been optimised to grow in a 2D environment and they cannot account for patient and tumour heterogeneity, therefore making personalised medicine impossible. Létourneau et al., developed new cancer cell lines in their study and had shown that there were differences in spheroid formation between cell lines derived before and after chemotherapy treatment [25]. This would be an important consideration when using cancer cell lines as the sensitivities shown in preclinical studies could be misinterpreted.

The focus of this review is to explore the existing knowledge base and evidence with regards patient derived samples of OvCa cells for fabrication of a 3D model to study the properties in vitro.

## 2. Materials and Methods

This review subscribes to the new PRISMA (Preferred Reporting Items for Systematic reviews and Meta-Analyses) guidelines [128].

### 2.1. Search Strategy

The eligibility criteria were studies published in English language and there were no other limitations to the search. Thorough literature search was performed using electronic databases of MEDLINE, EMBASE, Library, Information Science & Technology Abstracts, British Library Document Supply Centre Inside Serials & Conference Proceedings, ScienceDirect, Oxford Handbooks Online, Academic Search Index, Supplemental Index, Complementary Index, Directory of Open Access Journals, British Library EthOS, Digital Access to Scholarship at Harvard (DASH), University Press Scholarship Online, BioOne Complete, Center for Research Libraries, Research Starters, Oxford Medicine Online, Cochrane Database of Systematic Reviews, Oxford Bibliographies, Gale OneFile: Health and Medicine, Kent, Surrey and Sussex NHS Libraries, Springer Protocols, McGraw-Hill Medical, VleBooks, Kortext eBook Catalogue, BMJ Best Practice, ClinicalTrials.gov, Emerald InsightNCI (National Cancer Institute at the National Institutes of Health) (Bethesda, MD, USA), Directory of Open Access Books by two reviewers to identify relevant studies. Bibliographies of the relevant literature were also screened for any additional studies that were missed through the electronic search. Additional searching of the grey literature has also been conducted.

The exact syntax of search terms included ‘scaffold’, ‘three-dimensional’, ‘3D’ and ‘spheroid’ each of which were combined with term ‘ovarian cancer’. These terms were used as historically there has been interchangeability between the terms of “3D models” and “scaffolds”. All search terms were expanded, and all sub-categories were included. Thesaurus search was also used to identify additional terms. All duplicates were removed. The search was independently run by two of the authors. Databases last accessed 5 October 2022. The search protocol available on demand.

### 2.2. Selection Process

Initial screening of article headings was performed identifying the potential studies that could be used in current review. The selected studies were further screened using the abstracts and the irrelevant ones were excluded. The full text of these potentially eligible studies was retrieved and assessed for eligibility. Any discrepancies over the eligibility of particular studies were resolved through discussion with two other reviewers.

### 2.3. Study Selection

The studies included in the current review meet the following criteria:

- The type of cells used for the 3D model were exclusively patient derived OvCa cells or newly established cell lines derived directly from primary OvCa cells.
- Only multicellular tumour spheroid and tumour-derived spheroid models were reviewed in the current study.
- The main focus of the research was to build a three-dimensional model of OvCa cells regardless of method used to accomplish it.

Studies that used established OvCa cell lines or other types of cancer cells; organotypic multicellular spheroid and tumour derived organoid models and studies that used 3D modelling in their research but did not mention methods of construction were not included in the current review.

### 2.4. Data Extraction

Data was extracted from the selected papers using pre-designed data collection forms. Attempts were made to contact authors where data was missing from the papers.

## 3. Results

Following the electronic database search 3839 articles were identified and 2844 articles remained after removal of duplicates. Title screening resulted in rejection of 2435 papers as they did not meet the inclusion criteria. Out of the remaining 409 articles 326 were excluded based on abstract review.

Full manuscripts of all the remaining papers were reviewed. Following the screening of the bibliographies of the full manuscripts two additional papers were also reviewed.

The exclusion of the papers after abstract and full manuscript review was based on the following criteria:

- established OvCa cell lines were used for creating tumour spheroids opposed to patient derived samples;
- not OvCa cells used;
- animal models studied;
- only conference abstract was available which did not contain enough information for detailed analysis;
- review article;
- 3D model construction was described in the previous study of the same group, sensitivities were evaluated in the article;
- in vivo formed spheroids were the focus of the research;
- topic was found irrelevant.

Twenty-five full papers and eleven conference abstracts were included in the review.

**Figure A2:**
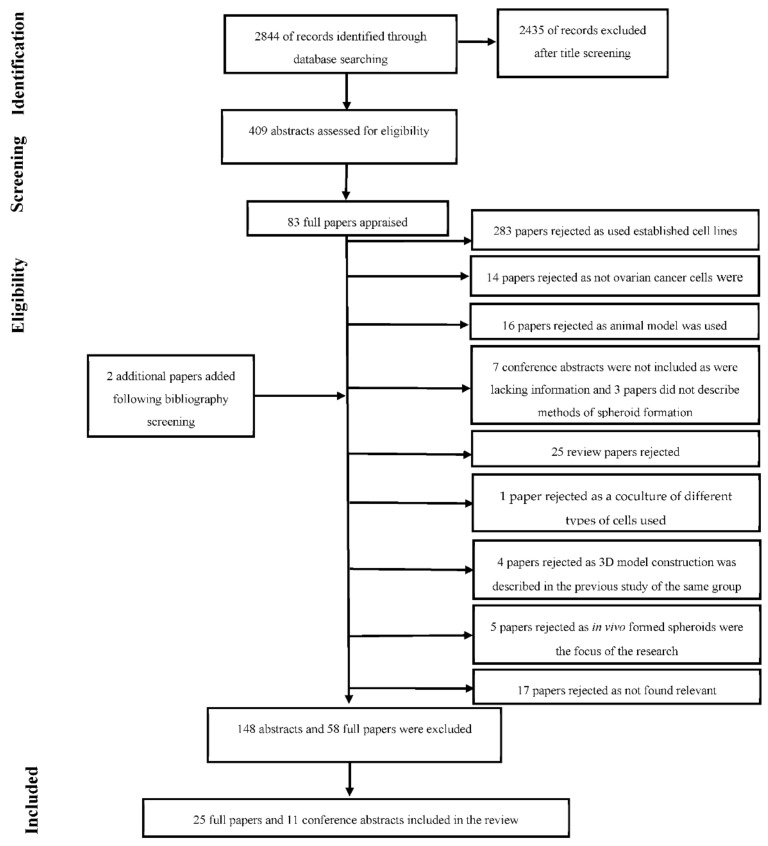
PRISMA flow chart.

Figure A2 in the Appendix A shows the full summary of the search and the process of the paper selection.

## 4. Discussion

The vast majority of the studies on OvCa models use in their research well-established cell lines, which in itself becomes a significant limitation as we discussed above. In this review we would like to focus on the papers that have used patient-derived samples to create OvCa models.

The relevant reports on 3D models for OvCa have been overviewed in Table 2, Table 3, Table 4, Table 5, Table 6 and Table 7 and will be further discussed herein.

Table 2 summarised the papers where fresh OvCa specimens were used to create 3D OvCa models along with the conference abstracts (Table 7), where all the authors used fresh specimens. Various samples have been used—solid tumours, biopsy specimens, ascites and pleural effusions. In some cases, the patients were chosen specifically to be chemotherapy naïve [8,17]. As evident from Table 2 and Table 7, in most of the cases if fresh tumour specimens were obtained, mincing and enzyme digestion was used to isolate cells for further cultivation.

To our knowledge the first study to utilise human tissue for construction of 3D ovarian tumour model was reported by Griffon et al., who were able to demonstrate spheroid growth in situ in eight cell suspensions out of eighteen, harvested from eight solid tumours, nine ascitic fluids and one pleural effusion. They noted that effusions were more productive in spheroid formation, than solid tumours and that samples obtained from mucinous ovarian adenocarcinomas did not form spheroids at all. [6]

Other studies have been able to establish new cell lines derived directly from fresh tumour specimens to further study the spheroid formation (Table 3). Again, surgical specimens and ascites of OvCa patients were used, however other methods of obtaining the material were described, like scrape method [23,25] and Grun et al., used cytobrushings of squamous ovarian carcinoma to establish a cell line [24]. In their study Puiffe et al., described spheroid formation using OV-90 cell line; as well as the effects of acellular fraction of patient-derived ascites on the spheroid formation, growth and invasion [22].

It becomes evident from the analysis of Table 2, Table 3 and Table 7 that more recently there is an ongoing trend towards utilising patient derived material to study tumour microenvironment and its molecular and biochemical features [9,10,11,28,29,30].

**Table 2:** Summary of papers, which used primary patient specimens to develop spheroid models.

Paper	Number of Patients	Type of Specimens Collected
Griffon et al. [6]	18	8 solid tumours finely chopped and enzymatically disaggregated, 9 ascitic fluids and 1 pleural effusion
Zhang et al. [7]	5	Tumour specimens of stage III serous adenocarcinomas—minced and enzymatically disaggregated
Kryczek et al. [8]	25	Cells and tissues obtained from ascites and tumours of chemotherapy naïve patients with EOC
He et al. [12]	6	Tumour specimens from OvCa patients mechanically dissociated and enzymatically disaggregated within 30 min of surgery
Martinez- Serrano et al. [13]	10	Ovarian tumour mass from chemotherapy naïve patients with papillary serous EOC processed using enzymatic cell tissue dissociation
Rafehi et al. [14]	At least 4 independent patient samples and at least 3 experimental replicates	Ascites fluid obtained from OvCa patients at the time of debulking surgery or paracentesis
Raghavan et al. [15]	3	Primary patient ascites cells (centrifuged) from tumour bank with confirmed OvCa origin
Loessner et al. [16]	n/a	Primary OvCa cells isolated from patients with high grade serous OvCa
Shuford et al. [17]	92	Fresh tissue from either a primary debulking surgery (n = 76) or laparoscopic biopsy (n = 16) of chemotherapy naïve patients
Maru et al. [18]	15	Tissue fragments of approximately 500–1000 mm^3^ obtained from ovarian tumours immediately after tumour resection. Non-necrotic lesions with solid or papillary growth selected. Tissue fragments cut into 2–3 mm pieces and enzymatically disaggregated.
Nelson et al. [19]	12	Primary patient ascites cells (centrifuged) from tumour bank and solid tumour samples processed using a tumour dissociation kit
Park et al. [9]	3	Fresh tumours minced and dissociated with collagenase
Huang et al. [10]	7	Fresh tumours minced and dissociated with collagenase and hyaluronidase
Hedemann et al. [11]	n/a	Fresh tumour cleared, fragmentated and enzymatically disaggregated

**Table 3:** Summary of papers, which used cell lines derived directly from primary patient specimens to develop spheroid models.

Paper	Number of Patients	Type of Specimens Collected
Sonoda et al. [20]	n/a	OVMG-1 and OVMG-2 serous adenocarcinoma cell lines from surgical specimens
Zietarska et al. [21]	n/a	TOV-21G and TOV-112I cell lines from primary ovarian malignant tumours; OV-90 cell line from ovarian malignant ascites from chemotherapy naïve patients
Puiffe et al. [22]	OV-90 cell line derived from 1 patient; ascites of 54 EOC patients	OV-90 cell line derived from cellular fraction of ascites from a chemotherapy-naïve patient
Ouellet et al. [23]	2	TOV-1946 (scape method used) and TOV-2223G (collagenase method used) cell lines derived from solid tumours and OV-1946 cell lines—from a mass of cells from ascites (micro-dissection into small pieces) of chemotherapy naïve patients with grade 3 serous papillary cystadenoma at stage IIIC
Grun et al. [24]	n/a	OV-TRL12B cell line established from cytobrushing of a squamous ovarian carcinoma
Létourneau et al. [25]	3	TOV cell lines (n = 4) derived from solid ovarian tumour (scape method) and OV cell lines (n = 5) established from the cellular fraction of ascites (centrifugation)
Liao et al. [26]	30	Primary EOC cell lines obtained from tumour specimens (finely minced) and ascitic fluid (centrifugation) obtained from patients undergoing tumour debulking surgery for EOC
Fleury et al. [27]	6	Solid ovarian tumour (TOV) derived cell lines (TOV2978G, TOV3041G, TOV3291G) (scrape method). The OV cell lines (OV866(2), OV4453, OV4485) established from the cellular fraction of ascites (centrifugation).
Noguchi et al. [28]	1	NCC-cOV1-C1 cell line derived from cellular fraction of ascites of a patient with clear cell carcinoma
Silva et al. [29]	1	IPO43 cell line established from the ascitic fluid of a patient with a diagnosis of high-grade serous carcinoma (HGSC) of the ovary, previously treated with chemotherapy
Parashar et al. [30]	1	Ovarian tumour samples minced and enzymatically disaggregated. Cells strained and centrifuged.

It is also evident from the literature that spheroid models are the most widely used systems for 3D in vitro models of OvCa, although few groups have assessed complex systems like hydrogels. Amongst the spheroid models, forced floating technique using commercially available specialised spheroid forming ultra-low attachment dishes is the most common method [5,6,7,8,10,11,12,14,17,20,22], followed by hanging drop method [15,21,23,25,27], all in static culture. A key difficulty of static 3D culture is their long-term maintenance, which is evident from the literature wherein most spheroid models are maintained for 24 h–7 days. Very few studies have been able to investigate long term culture of spheroid models. For example, some authors maintained their model for 2 weeks [7,12,13,16] while Kryczek et al., went further and investigated the spheroids in the culture for 6 weeks [8].

Grun et al., established spheroids in Rotary Cell Culture System and highlighted that the used method had allowed spheroids to grow for longer periods and reach significantly greater ‘tumour’ volumes than when using hanging droplet method. Although in their study authors were able to reach a maximum diameter of 4 mm, extensive areas of necrosis were present [24]. Loessner et al., described an encapsulation-based spheroid formation using a hydrogel system, which allowed them to create a 3D culture showing cell proliferation and aggregation similar to in vivo. The method and the 3D system were optimised using cell line OVMZ-6 and then used for primary patient samples to establish that their method worked on primary samples [16]. Finally, Maru et al., used hydrogel-based sandwich method with incubation time of 5 days to create the 3D model. Their 3D hydrogel-based model of patient samples was able to maintain original tumour characteristics. However, they found a significant limitation of this method for gynaecological tumours, as a result of which insufficient number of cells were able to attach to hydrogel platform [18] The time of incubation ranged from minimum of 24 h [17] to 6 weeks [8] with most of the studies validating the findings in vivo.

**Table 4:** Summary of methods used to originate 3D structures from patient derived cells.

Hanging Drop	Forced Floating	Bioreactor	Others
Zietarska et al. (4 days)	Griffon et al. (4–5 days)	Grun et al. (3–4 weeks)	Loessner et al. (hydrogel system; 2 weeks)
Ouellet et al. (3 days)	Sonoda et al. (7 days)		Maru et al. (hydrogel-based sandwich method; 5 days)
Létourneau et al. (4 days)	Puiffe et al. (4 days)		
Fleury et al. (5–7 days)	Zhang et al. (11–14 days)		
Raghavan et al. (7 days)	Kryczek et al. (1–6 weeks)		
	Liao et al. (3 weeks early culture- > dissociation and replating fornightly)		
	Rafehi et al. (72 h)		
	He et al. (14 days)		
	Martinez-Serrano et al. (average 28 days)		
	Shuford et al. (24–72 h)		
	Nelson et al. (2–4 days)		
	Vader et al.		
	Basten et al.		
	Mikkonen et al.		
	Nanki et al.		
	Park et al. (7 days)		
	Noguchi et al. (4 days)		
	Hedemann et al. (4 days)		
	Huang et al.		
	Silva et al. (72 h)		
	Parashar et al. (7 days)		

The biggest size of spheroids was demonstrated by Grun et al., who used the Rotary Cell Culture System to culture them [24], whereas others grew spheroids with a maximum size up to 500 µm [21,22] and the smallest of 50–100 µm by Zhang et al. [7]. Zietarska et al., reported an absence of hypoxic or necrotic cores, which they related to relatively short culture time (4 days) as well as small size of spheroids [23].

One of the key findings of Kryczek et al., was that deletion of ALDH+ cells or CD133+ cells dramatically reduced the quantity and size of spheres formed, whereas their simultaneous deletion drastically reduced sphere formation. Furthermore, they were able to show that the expression of ALDH+ and CD133+ gradually reduced following prolonged in vitro cycles [8]. Other studies have gone down the path of comparing the 2D and 3D systems [20,21,24]. Sonoda et al., looked at the expression of VEGF, IL-8 etc., which was compared between monolayer, spheroids and animal model [20]; Zietarska et al., following cluster analysis of gene expression suggested differences amongst the three types of models, such as the expression of *THBS1*, *PECAM1* genes and others [21]; Grun et al., carried-out comparison between 2D and 3D culture in terms of proteomic profiling and observed differences between the two for various markers including those of proliferation, apoptosis (CA125, BCL2, proliferation marker, Mib-1, p53, CK7 and others) [24]. Another study looking at the molecular effects of spheroids, reported that spheroid formation promoted/induced epithelial to mesenchymal transition (EMT) for EOC cells which was decreased on re-attachment of the spheroids. They also observed increased expression of TGFβ 1 in EOC spheroids, which they hypothesis modulates EMT in spheroid cultures [14].

A lot of the studies aimed to look at the cellular characteristics of the established spheroids. For example, it was shown by Puiffe et al., that compact spheroids were able to form by the cell line culture in the presence of patient derived acellular fraction of ascites suggesting the importance and subtleties of ascites in modulating the tumour microenvironment. They also studied the effects of ascites on invasion, proliferation and gene expression in the developed spheroids [22]. The same authors developed cell lines from patient samples and characterised different phenotypic characteristics including their ability to form 3D tumour models as spheroids. For instance, the authors showed that only TOV-112D cell line was able to form compact spheroids when hanging droplet method used, whereas other cell lines either formed cell clusters or did not show any aggregation properties at all [23]. Similar to these group, other authors also developed new cell lines and tested the ability of those to form 3D structures [25,26,27]. Liao et al., were able to isolate spheroids that were tumorigenic in vivo and had higher proliferation and migration in comparison to their parent non spheroid cells; they also observed a difference in expression of various stem cell markers, like Notch1, Nanog, D34 etc., higher than non-spheroid cells under same growth conditions [26]. Fleury et al., in their study had also shown that different cell lines derived from patient samples had different mutations seen in EOC cells (TP53, BRCA1, BRCA2 etc.) [27]. From the studies using patient derived material, Loessner et al., established the importance of co-culture system involving OvCa and mesothelial cells [16]; Zhang et al., described the process of isolation and characterisation of highly tumorigenic subpopulation of cells (malignant progenitors) [7]; whereas Maru et al., showed that 3D hydrogel-based model of patient samples was able to maintain original tumour characteristics. They also inferred that spheroid-based models are better for assessment of treatments in comparison to hydrogel-based 3D in vitro models [18].

All of the above studies confirm the benefits of 3D models in the investigation of the tumour characteristics, microenvironment and its ‘behavior’ and as it was highlighted by Maru et al., the choice of 3D system should be dependent on the end goal/objectives [18]. A key takeaway point from the various publications involving 3D models of OvCa is their similarity to in vivo conditions. For example, Sonoda et al., showed that VEGF expression was enhanced in spheroid models in comparison to 2D monolayer of the same cell lines. [20] Gene expression analysis carried out by Zietarska et al., identified genes in 3D model which mimic in vivo tumour gene expression in contrast to 2D culture wherein such genes were not expressed [21]. It has also been reported by Raghavan et al., that spheroid models were able to mimic A2780 and OVCAR3′s in vivo characteristic of resistance to cisplatin, which is not seen in 2D in vitro culture [15]. These aspects once again highlight the need for the research community to move towards a 3D model-based approach in comparison to 2D systems.

The similarity to in vivo conditions of the patient derived 3D culture has a major advantage of helping in treatment studies for cancer patients. The first study to utilise this was by Griffon et al., who assessed the radiosensitivity (0–8 Gy) of primary OvCa cells from various locations using tumour spheroid model. They observed extensive variation between 3D spheroids from different patients in terms of their response to radiotherapy, highlighting the need for personalised treatment protocol for patients in a clinical setting [6]. Similar studies were conducted to assess chemosensitivity of OvCa cells in 3D spheroid models by Zhang et al. They reported higher resistance to cisplatin and paclitaxel for 3D spheroids with stem cell like properties in comparison to differentiated ones, suggesting a plausible reason behind OvCa’s high recurrence rate [7]. Liao et al., along with He et al., who had similar findings, showed that spheroid forming cells that maintained stem cell like properties were more resistant to cisplatin in comparison to their parent cells [12,26]. More recent studies have been testing patterns of chemoresistance [15] and therapy response predictions [17] on in vitro models. Raghavan et al., used cells recovered from primary patient malignant ascites by centrifugation and demonstrated differences in therapeutic response between patient-derived samples as well as showed correlation with in vivo drug studies in xenografts [15], whereas Shuford et al., conducted a large study including 92 samples of fresh tissue from either a primary debulking surgery or laparoscopic biopsy of chemotherapy naïve patients [17]. Looking at the abstracts included in this review it also becomes evident how important it is becoming in the recent years to create a 3D model for treatment prediction as one of the key highlights of 3D systems is that they mimic the effect of therapy better than 2D. Multiple study reports show differences in sensitivities of drug agents used for treatment of OvCa, such as carboplatin [17,34,41], cisplatin [40,127], paclixatel [33,40,41,127] and others [34,35,36,37,38]. Details of each of the studies included in this review could be seen in Table 5, Table 6 and Table 7.

These reports have shown the most recent advances in personalised approach to treatment prediction in OvCa patients, however it is still evident from this analysis that further research is required of patient derived studies in 3D due to their obvious advantages for investigation of tumour characteristics and more importantly better and personalised drug screening.

The vast majority of the 3D in vitro models developed for OvCa studies are spheroid type models and hydrogel types of models. Despite advantages of spheroids (especially compared to 2D cultures), such as simplicity of fabrication, achievement of heterogeneity in phenotype and gene expression and altered cell metabolism they do have several limitations. Specifically, there is an exceedingly high variability of their aggregate forming densities, which is also supported in the most recent research papers [11,30], they are susceptible to dissociation during handling and experimentation, crucial tumour microenvironment (TME) conditions (ECM, cell-matrix interactions, stiffness and mechanical properties) cannot be controlled, they cannot recapitulate in vivo mass transfer limitations, vascularisation does not occur, and they cannot be cultured long term [10]. For instance Hedemann et al., had shown, that the spheroids grown from primary cancer cells were not able to exhibit the same degree of growth as cell-lines in the same “environment” conditions [11]. Hydrogels, based on their chemistry are more advanced than spheroids, offering some level of structure, ECM mimicry and porosity, however, they have low mechanical strength and artificially high-water content (some of them up to 95%), which leads to an unrealistic microenvironment for the cells. Moreover, although generally hydrogels depending on their porosity and pore inter-connectivity could mimic various densities of different tissues, they lack other components present in connective tissue; whereas while Matrigel resembles the laminin/collagen IV-rich basement membrane extracellular environment, it does not accurately mimic the basement membranes [22].

Finally, as it is evident from the review there are currently no studies that have been able to create OvCa polymeric scaffold 3D model utilising patient-derived cells, which would significantly improve patient care by predicting the efficacy of potential chemotherapy treatment and be a further step forward into investigating the microenvironment and biology of EOC.

**Table 5:** Summary of papers, which used primary patient specimens to develop spheroid models—part 2.

Paper	Construct Development Method		Time of Incubation		Size of Spheroids	General Comment
Griffon et al. [6]	6-well plates coated with 1 mL of 0.5% agarose (forced floating/aggregation)		10 days in vitro		Mean of 198 (±7.7) µm	Radiosensitivities of spheroids obtained from human ovarian carcinoma cells tested.
Zhang et al. [7]	Ultra Low Attachment plates (forced floating/aggregation)		11–14 days in vitro	Validation in vivo	50–100 µm	Isolation and characterisation of highly tumorigenic subpopulation of cells (malignant progenitors) described.
Kryczek et al. [8]	Ultralow attachment plates	(forced floating/aggregation)	Spheres counted for 1–6 weeks	Validation in vivo	>50 µm	Expression of multiple cancer stem cell markers in fresh OvCa and established primary OvCa cell lines investigated and the stem cell properties of potential OvCa stem cells in vitro and in vivo examined.
He et al. [12]	96-well ultra-low attachment plates	(forced floating/aggregation)	2 weeks in vitro	Validation in vivo	n/a	Subpopulation of stem cell-like cells that form spheroids and possess self-renewal capacity, strong tumour-initiating ability, and higher resistance to chemotherapy derived from high grade serous carcinoma studied.
Martinez-Serrano et al. [13]	Corning Ultra-Low attachment surface T25 flask	(forced floating/aggregation)	Median period of 28 days in vitro cultivation		n/a	Specificity of cell surface markers to discriminate the tumour initiating cells (isolated from EOC) from somatic stem cells (isolated from healthy women) investigated.
Rafehi et al. [14]	Ultralow attachment plates	(forced floating/aggregation)	3 days in vitro		n/a	Findings demonstrating that intact TGFβ signalling is required to control epithelial-mesenchymal transition in EOC ascites-derived cell spheroids, and it promotes the malignant characteristics of these structures.
Raghavan et al. [15]	Spheroids formed by hanging drop method		7 days in vitro	Validation in vivo	n/a	The responses to varying drug treatments were different in patient-derived samples and correlated with in vivo drug studies in xenografts.
Loessner et al. [16]	Encapsulation based spheroid formation		2 weeks in vitro		n/a	3D culture showed cell proliferation profile and aggregation similar to in vivo. Expression of integrins, MMP enhanced in 3D culture in comparison to 2D. Spheroids showed higher chemoresistance in comparison to 2D for paclitaxel.
Shuford et al. [17]	84-well spheroid microplates	(forced floating/aggregation)	24 h in vitro		n/a	Analytical and prospective clinical validation of a new test that utilizes primary patient tissue in 3D cell culture to make patient specific response predictions prior to initiation of treatment in the clinic presented.
Maru et al. [18]	Hydrogel based sandwich method		5 days in vitro		n/a	3D hydrogel-based model of patient samples was able to maintain original tumour characteristics. Spheroid based models are better for assessment of treatments in comparison to hydrogel-based 3D in vitro models.
Nelson et al. [19]	Matrigel in 24-well plate (forced floating/aggregation)		2–4 days in vitro		n/a	Ex vivo cultures from patient biopsies used to provide models that support interrogation of chromosome instability mechanisms.
Park et al. [9]	Ultra-attachment 6-well culture plates	(forced floating/aggregation)	7 days in vitro		n/a	mRNA expression of transcription factors and miRNA expression of spheroids derived from primary ovarian cancers to identify factors regulating ovarian cancer stem cells.
Huang et al. [10]	6-well ultra-low attachment plates. (forced floating/aggregation)		7–10 days in vitro	Validation in vivo	>50 µm	Cell lines and primary tissue used to grow spheroids, which were tested against platinum-chemotherapy agents, correlated with in vivo drug studies in xenografts.
Hedemann et al. [11]	Ultralow attachment plates	(forced floating/aggregation)	4 days in vitro		~150–300 µm	A combination of ADAM17 inhibitor with cisplatin tested in 2D and 3D culture of cells derived from cell lines and primary ovarian tumor- and ascites-derived cells.

**Table 6:** Summary of papers, which used cell lines derived directly from primary patient specimens to develop spheroid models—part 2.

Paper	Construct Development Method		Time of Incubation		Size of Spheroids			General Comment
Sonoda et al. [20]	24-well culture plate coated with 1% agarose (forced floating/aggregation)		7 days in vitro	Validation in vivo	n/a			Angiogenesis factors expression measured and compared in 2D, 3D and xenografts.
Zietarska et al. [21]	Spheroids formed by hanging drop method		10 days in vitro (spheroids formed by day 4)	Validation in vivo	Maximum size of 500 µm			Molecular comparison of spheroid model versus 2D and xenograft model described.
Puiffe et al. [22]	Spheroids formed by modified hanging drop method		4 days in vitro		Small in the absence And ~500 µm in the presence of ascites			Effect of the acellular fraction of ascites on OV-90 addressed.
Ouellet et al. [23]	Spheroids formed by modified hanging drop method		4 days in vitro	Validation in vivo	TOV-1946—aggregate	OV-1946—semi-compact	TOV-2223—none	New serous EOC cell lines from both solid tumours and ascites of the same patient were derived and characterised.
Grun et al. [24]	Rotary Cell Culture System		3–4 weeks in vitro (spheroids formed in 1 week)		Maximum diameter of 4 mm			3D culture established, biological features (morphological characteristics, expression of tumour markers, proteomic profiles). compared between 2D, 3D and primary tumours.
Létourneau et al. [25]	Spheroids formed by hanging drop method		4 days in vitro	Validation in vivo	n/a			New OvCa cell lines described.
Liao et al. [26]	Ultralow attachment plates	(forced floating/aggregation)	Cultivation period n/a	Validation in vivo	n/a			To study EOC pathogenesis, EOC primary cells under stem cell selective conditions were cultured and generated anchorage-independent, self-renewing spheroids morphologically similar to spheroids isolated from patient ascites.
Fleury et al. [27]	Spheroids formed by hanging droplet method		5–7 days in vitro	Validation in vivo	TOV2978G, OV4453—aggregate	OTOV3291G—semi compact	TOV3041G—compact	Six new EOC cell lines spontaneously derived from high grade serous tumours or ascites established and described.
Noguchi et al. [28]	96-well culture plates (forced floating/aggregation)		4 days in vitro		n/a			NCC-cOV1-C1 cell line established and characterised. Anticancer drug screening conducted.
Silva et al. [29]	Stirred-tank culture system placed on a magnetic stirrer		3 days of in vitro		n/a			IPO43 cell line established and characterised.
Parashar et al. [30]	24-well, growth factor reduced Matrigel-coated non-adherent plates (forced floating/aggregation)		7 days in vitro		~30–100 µm			MCW-OV-SL-3 endometrioid subtype of ovarian cancer cell line established, chemoresistance mechanisms studied.

**Table 7:** Summary of conference abstracts.

Abstract	Year	Number of Patients, Specimen and Method	General Comment
Sun et al. [31]	2012	Fresh specimens of OvCa minced, enzymatically digested, rinsed, incubated in DMEM (monolayer cultures) Mammosphere media (spheroids). Validation in vivo.	Spheroids are enriched for expression of markers including CD133, CD44, NANOG and OCT4, suggesting that spheroid formation enhances stem cell-like markers. Increased expression of miR-26b in spheroids compared to monolayer culture.
Shuford et al. [32]	2014	OvCa samples—standard mincing & digestion.	Ex vivo 3D (EV3D™) culture and testing of primary human OvCa was described. Carboplatin & taxane based combination therapy was used in most cases.
Ishiguro et al. [33]	2014	OvCa cells from surgical specimen. Validation in vivo.	Differentiation of spheroid cells associated with the downregulation of the stem cell-specific regulators Nanog, Sox2, and ALDH1A1 and the up-regulation of cytokeratin and it is associated with increased paclitaxel resistance. The changes are reversible.
Desrochers et al. [34]	2015	OvCa samples (newly diagnosed, treatment naïve and relapsed) standard mincing & digestion. 3D spheroids were developed and 3D perfused Ovarian Microtumours were cultured using the 3DKUBETM.	Carboplatin, gemcitabine, erlotinib and afatanib responses tested.
Vader et al. [35] Basten et al. [36] Dijkmans et al. [37]	2017 2018 2018	3D cultures embedded in a protein-rich hydrogel (384 well plates) are generated from tumour biopsies (endometrial, cervical, and OvCa patients–fresh and cryopreserved material).	3D cultures exposed to standard-of-care therapies, targeted therapies and drug combinations.
Mikkonen et al. [38]	2018	Processed fresh cancer tissue (ovarian)—cells cultivated in Matrigel or in cellulose-based hydrogel, GrowDex.	Genetic profiling and image-based phenotyping, phenomics done. Drug responses (52 agents) tested in 2D and 3D, significant differences in sensitivity to several drugs observed.
Nanki et al. [39]	2018	Intraoperative ascites and tissue samples from primary ovarian, peritoneal, and fallopian tube cancer patients. 3D culture obtained using 96-well plates—14 days.	Spheroids-like structures were formed in 30% (1/3) of ascites samples and 50% (4/8) of tissue samples. The tumorigenicity and invasiveness of the cells were demonstrated using new 3D model cultured in vitro by NanoCulture Plate LH96.
Tanaka et al. [40]	2018	13 primary ovarian tumour surgical samples (8—OvCa, 2—borderline, 3—benign) and 1 malignant effusion (ascitic and pleural) of OvCa patient. Matrigel-based organoid culture, or spheroid culture.	Long-term 3D cultures established from 4 samples. Drug responses tested for 2 cultures (cisplatin and paclitaxel).
Ito et al. [41]	2018	OvCa cells from patient tumours (61 cancer tissue-originated spheroid (CTOS) method).	Sensitivity assay for paclitaxel and carboplatin conducted and compared to clinical outcome.

## 5. Conclusions

There is a clear need for the development of an accurate, robust, 3D system which will enable the culture and drug screening of patient derived ovarian tumours. Such a system will allow screening of drugs as well as genetic analysis of the cancer of a specific individual, therefore, optimising/tailoring the treatment towards that individual. Furthermore, such an in vitro 3D system which would account for the tumour microenvironment heterogeneity, would help elucidate developmental and evolutionary aspects of the disease. Finally, for the development of such system with a tangible clinical outcome a systematic rigorous experimentation with patient derived tumours (and not with cell lines) is essential.

While this review covers studies of 3D OvCa models utilising patient derived samples, we have limited ourselves to multicellular tumour spheroid and tumour-derived spheroid models; and the review focused mainly on the models of EOC.

## Figures and Tables

**Table 1:** Key characteristics of an ideal model of ovarian tumour.

Key Element/Characteristic of Ovarian Tumour Model	In Vivo Function/Repercussion
Complex microenvironment (cellular and architectural) [42,43,44,45]	• Reflects tumour histology • Tumour growth • Resistance to chemotherapeutic agents
Mesothelial cells [43,47]	• Attachment and invasion of cancer cells
Fibronectin/Integrins [43]	• Spheroidal structure growth
Fibroblasts [52]	• Tumour growth, adhesion and invasiveness
Adipocytes [49,50,51]	• Tumour growth and metastasis promotion
Extracellular matrix and stroma [51]	• Tumour growth, adhesion
Extracellular microvesicles [47]	• Invasion and methastasis • Drug-resistance
Angiogenesis (PARP/VEGFR3)/Neovascularisation [54,55,59]	• Ability to grow over a certain size • Invasion and metastasis • Drug-resistance
Ability to self-organise in 3D structures [43]	• Invasion and metastasis • Drug-resistance

## Data Availability

Data supporting reported results can be found on: https://search.ebscohost.com/login.aspx?direct=true&AuthType=sso&bquery=AB+scaffold+AND+TI+(+ovarian+cancer+or+ovarian+neoplasms+)+OR+AB+3d+AND+TI+(+ovarian+cancer+or+ovarian+neoplasms+)+OR+AB+three+dimensional+AND+TI+(+ovarian+cancer+or+ovarian+neoplasms+)+OR+AB+spheroid+AND+TI+(+ovarian+cancer+or+ovarian+neoplasms+)&type=1&searchMode=And&site=eds-live&ssl=y&custid=ns124426 (accessed on 25 October 2022).

## Abbreviations

OvCa	ovarian cancer
EOC	epithelial ovarian cancer
3D	three dimensional
2D	two dimensional
TME	tumour microenvironment
ECM	extracellular matrix
PEG	poly-ethylene glycol
EMT	epithelial to mesenchymal transition
MMP	matrix metalloproteinases
CTOS	cancer tissue originated spheroids
TGFβ	transforming growth factor β
VEGF	vascular endothelial growth factor
